# Lack of Functional Selectin Ligand Interactions Compromises Long Term Tumor Protection by CD8^+^ T Cells

**DOI:** 10.1371/journal.pone.0032211

**Published:** 2012-02-16

**Authors:** Felicity C. Stark, Komal Gurnani, Subash Sad, Lakshmi Krishnan

**Affiliations:** 1 Department of Biochemistry, Microbiology and Immunology, University of Ottawa, Ottawa, Ontario, Canada; 2 National Research Council-Institute for Biological Sciences, Ottawa, Ontario, Canada; Saint Louis University School of Medicine, United States of America

## Abstract

Central memory CD8^+^ T cells expressing the adhesion molecule CD62L (L-selectin) are potent mediators of anti-cancer immunity due to their ability to proliferate extensively upon antigen re-stimulation. The interaction of selectin with its ligands mediates leukocyte rolling along high endothelial venules. Mice deficient in α(1,3) Fucosyltransferase IV and VII (FtDKO) lack functional L, P and E selectin ligands. Thus, we addressed whether the lack of selectin ligand interactions alters tumor protection by CD8^+^ T cells in FtDKO mice. Listeria monocytogenes-OVA (LM-OVA) infection evoked potent OVA-specific CD8^+^ T cells that proliferated and contracted at similar kinetics and phenotype in FtDKO and wild-type mice. Additionally, OVA-specific CD8^+^ T cells in both mouse strains exhibited similar phenotypic differentiation, in vivo cytolytic activity and IFN-γ expression. However, FtDKO mice succumbed to B16-OVA tumors significantly earlier than wild-type mice. In contrast, FtDKO mice evoked strong recall memory CD8^+^ T cell responses and protection to systemic LM-OVA re-challenge. The diminished tumor protection in FtDKO mice was not related to defective antigen presentation by dendritic cells or reduced proliferation of antigen-specific CD8^+^ T cells. However, WT or FtDKO OVA-specific CD8^+^ T cells showed significantly reduced ability to traffic to lymph nodes upon adoptive transfer into naïve FtDKO recipients. Furthermore, FtDKO OVA-specific CD8^+^ T cells displayed poor ability to infiltrate tumors growing in WT mice. These results reveal that selectin ligand expression on host endothelium as well CD8^+^ T cells may be important for their efficient and continued extravasation into peripheral tumors.

## Introduction

Vaccination represents a promising approach towards tumor eradication as CD8^+^ T cells play an important role in anti-tumor immunity [1]. Cancer antigen delivered by live bacterial and viral vectors such as recombinant Listeria monocytogenes, Salmonella enterica serovar Typhimurium and adenovirus have been effective at inducing strong CD8^+^ T cells against immunogenic tumors like metastatic melanoma [2]–[6]. Furthermore, adoptive transfer therapies involving tumor-antigen specific CD8^+^ T cells have shown cancer regression in clinical trials [1]. Moreover, memory CD8^+^ T cells can vary in both magnitude and quality [7]–[9] and generating a central memory population with a high level expression of CD62L (T_CM_) yields longer lasting tumor regression compared to CD62L low effector memory cells (T_EM_) [10]–[12]. We have previously reported that the choice of vaccine adjuvant and/or vector can also differentially impact the proportion of the two types of memory CD8^+^ T cells; for example Listeria monocytogenes generates a predominant CD62L^high^ central memory cells [13]. Nevertheless, clinical trials have revealed only a modest benefit of cancer immunotherapy and new approaches are required for objectively maximizing CD8^+^ T cell longevity, mobility, functionality and memory responsiveness [1].

CD62L (L-selectin) is a cell surface adhesion molecule found to be upregulated on naive and memory lymphocytes. It transiently interacts with selectin ligands on adjacent cells which initiate the tethering and rolling of lymphocytes along high endothelial venules, later leading to other firm adhesions that mediate entry of the lymphocyte into lymphoid tissue [14]. Selectin ligands are transmembrane glycoproteins that present the carbohydrate antigen, sialyl Lewis X to their cognate receptors, L,P and E-selectins [15], [16]. The synthesis of functional selectin ligands requires many post-translational modifications including a terminal fucosylation mediated by α(1,3) Fucosyltransferase IV and VII [17], [18]. Selectin ligand-selectin interactions may be important in many points in an immune response from the interaction of naive and memory T cells with high endothelial venules of lymph nodes to later interactions of activated T cells with activated epithelium at a site of inflammation. A redistribution of naive CD8^+^ T cells towards non-lymphoid organs occurs when selectin ligand interactions were interrupted [19], [20]. Since, it has been postulated that CD62L mediated trafficking of CD8^+^ T cells to lymph nodes may not be necessary for central memory formation [21], it is unclear whether selectin ligand interactions are necessary for the maintenance of an anti-tumor CD8^+^ T cell response.

We have used a tumor vaccine model, Listeria monocytogenes expressing Ovalbumin (LM-OVA) vaccination against the B16-OVA murine melanoma. CD8^+^ T cell responses and tumor protection were studied in wild-type (WT) C57BL/6J or α(1,3) Fucosyltransferase IV&VII-double deficient (FtDKO) mice lacking functional L-selectin ligand interactions. We show that FtDKO mice, similar to WT mice, develop a robust effector antigen specific CD8^+^ T cell response and memory with similar kinetics and recall ability against bacterial infections. Nevertheless, FtDKO mice exhibited reduced tumor protection against B16-OVA due to the differential trafficking ability of their CD8^+^ T cells.

## Results

### LM-OVA induced tumor immunity is compromised in FtDKO mice

**Figure 1:**
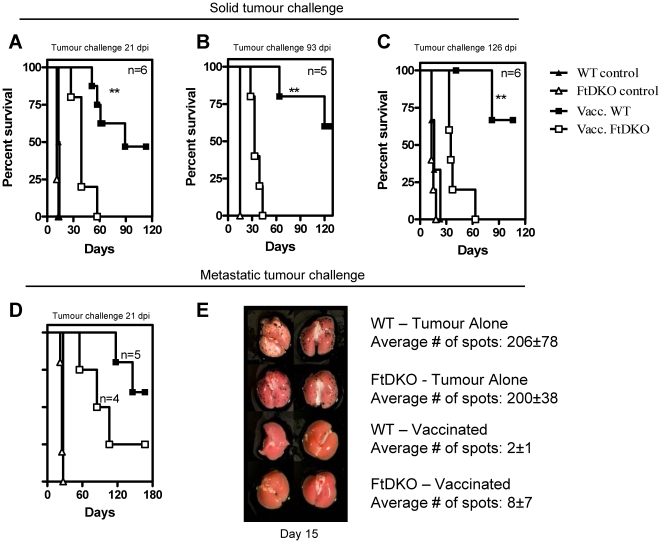
Prophylactic vaccination against B16-OVA/. C57BL/6J (WT) and FtDKO mice were vaccinated intravenously with LM-OVA and challenged with B16-OVA subcutaneously (A) 21 days, (B) 93 days or (C) 126 days post-infection. Survival curves were based on animals reaching a tumor size, 200 mm^2^. (D), Mice were challenged intravenously with B16-OVA, 21 days post LM-OVA infection. Survival was based on animals exhibiting a maximum weight loss of 20% or other clinical signs of illness. (E), Lung metastatic foci enumerated on day 15 post-tumor challenge in representative mice. Similar trend was noted for n = 5 mice/group. Significant differences were observed in panel A (p = 0.0082), panel B (p = 0.0015) and panel c (p = 0.03) by log-rank test.

Mice (WT and FtDKO) were vaccinated with LM-OVA and at days 21, 93 or 126 post-vaccination challenged with B16-OVA tumor cells. A significant delay in subcutaneous tumor development was observed in both FtDKO and WT vaccinated mice compared to non-vaccinated controls (Fig. 1A, B, C). However, regardless of the time of tumor challenge, all vaccinated FtDKO mice succumbed to subcutaneous tumors significantly quicker relative to vaccinated WT group (p<0.001, 0.0015, 0.003 for panels a, b and c respectively). In a metastatic model as well, longer survival after tumor challenge in vaccinated WT mice was observed relative to FtDKO mice (Fig. 1D); undefined median survival for the former compared to 95 days for the latter (p = 0.125). On day 15 after metastatic tumor challenge, vaccinated WT and FtDKO mice harbored similar number of black foci in the lungs, whereas vaccinated mice exhibited minimal tumor burden at this early time point (Fig. 1E). The tumor protection observed in vaccinated mice was antigen dependent as infection with LM did not afford protection (data not shown). These data indicate that B16-OVA tumor cells grew subcutaneously and metastatically at a similar pace in non-vaccinated FtDKO and WT mice. However, LM-OVA vaccinated FtDKO mice showed compromised tumor immunity.

### LM-OVA induces a potent CD8^+^ T cell response in FtDKO mice

**Figure 2:**
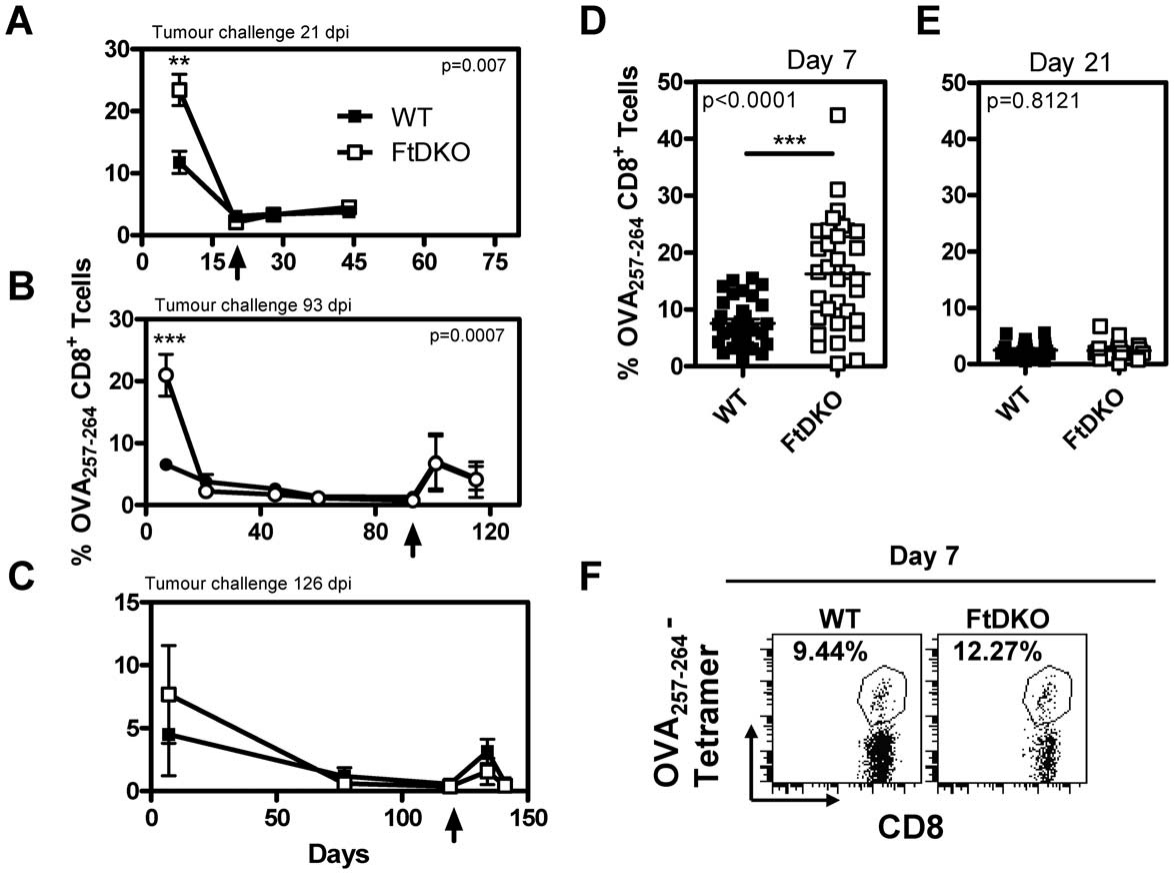
Frequency of OVA-CD8^+^ T cells following LM-OVA infection. Mice were infected with LM-OVA and challenged with B16-OVA subcutaneously 21 days (A), 93 days (B) or 126 days (C) post-infection (dpi). Tumor injection time point is indicated by an arrow on the x axis. The mean ± SD (n = 5 per group) of the frequency of OVA_257–264_-specific CD8^+^ T cells in the blood at various times is indicated (A, B, C). The frequency blood OVA_257–264_-specific CD8^+^ T cells on day 7 and 21 after LM-OVA infection in individual animals from seven different experiments is presented (D & E). Representative profile of blood OVA_257–264_-specific CD8^+^ T cell percentages (F). Response on day 7 is statistically different between the two mouse strains by two tailed, unpaired *t* test with Welsh's correction for unequal variance; **, P<0.001, ***, P<0.0001.

The frequency of OVA_257–264_-specific CD8^+^ T cells in the blood was tracked in independent experiments where animals were vaccinated with LM-OVA and later challenged with B16-OVA tumor cells on day 21 (Fig. 2A) day 93 (Fig. 2B) or day 126 (Fig. 2C). The frequency of OVA_257–264_-specific CD8^+^ T cells in FtDKO mice was significantly greater on day 7 post LM-OVA infection in 2 out of the 3 experiments. Further analysis of seven independent experiments, confirmed a higher frequency of blood OVA_257–264_-specific CD8^+^ T cells in FtDKO mice on day 7 post-infection (Fig. 2D). The CD8^+^ T cell contraction was also similar in both WT and FtDKO mice as the frequency declined from day 7 to 21 (Fig. 2E). Additionally, a low steady frequency of OVA_257–264_-specific CD8^+^ T cells was detectable in the blood (∼1%) up to 119 days post infection in both mouse strains (Fig. 2B & C). Upon tumor challenge a notable expansion of OVA_257–264_-specific CD8^+^ T cells occurred for both mouse strains. Therefore, upon LM-OVA vaccination, FtDKO mice exhibited no defect in CD8^+^ T cell priming, maintenance or recall response.

### LM-OVA induces a similar phenotype of CD8^+^ T cells in WT and FtDKO mice

**Figure 3:**
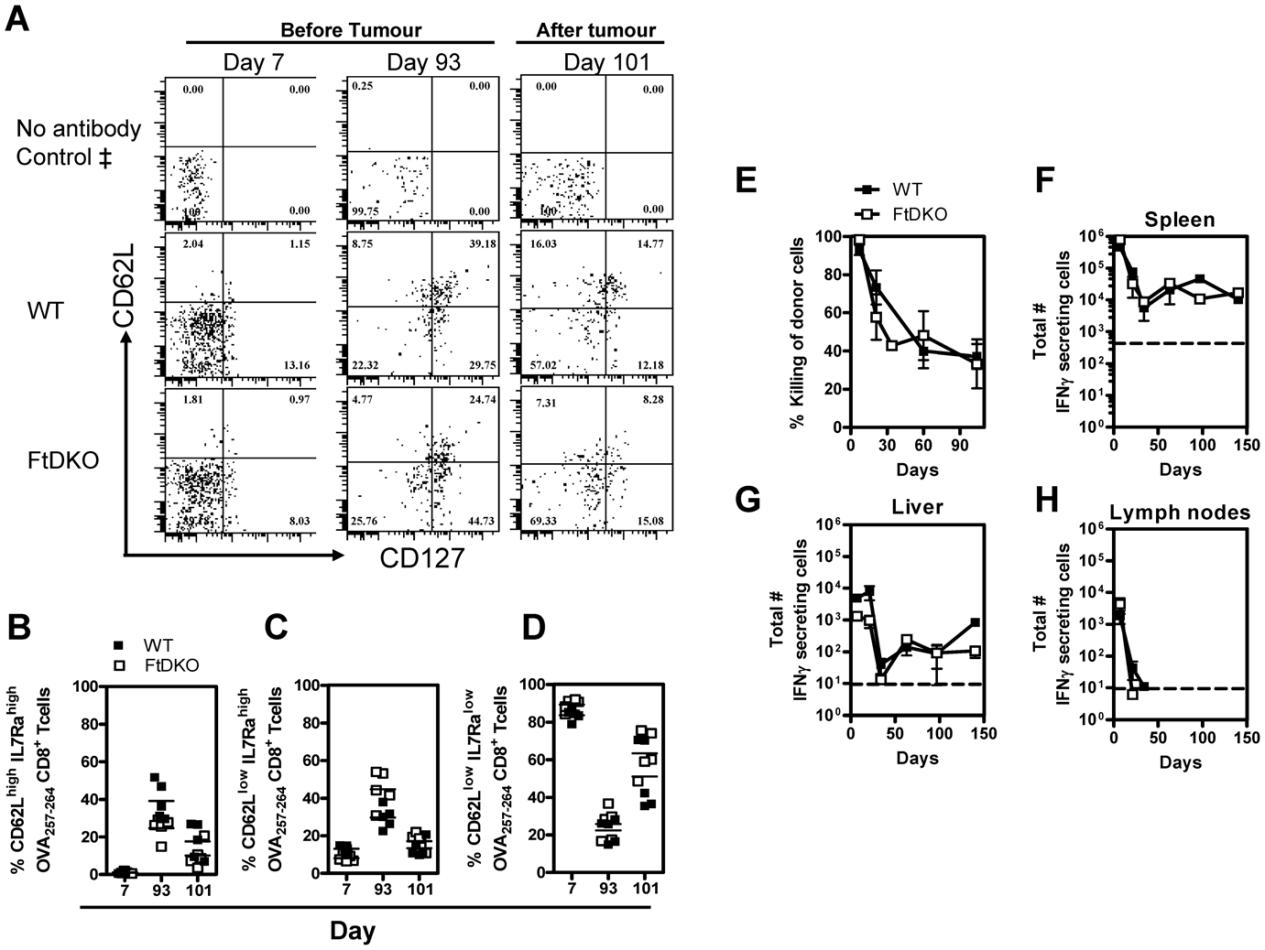
Phenotype and functionality of OVA-CD8^+^ T cells. C57BL/6J (WT) and FtDKO mice were immunized intravenously with 10^4^ LM-OVA. Representative flow cytometric profile indicating the expression of CD62L and IL-7Rα on OVA_257–264_-specific CD8^+^ T cells on day 7 and 93 and 101 (8 days after tumor challenge) after infection (A). ‡, Fluorescence minus one (FMO) controls was used to set the gates for IL-7Rα and CD62L. ‘No antibody control’ indicates gating strategy based on acquisition of cells stained with anti-CD8 antibody and OVA_257–264_ tetramer but not IL-7Rα or CD62L antibody. The frequency of T_CM_ (B), T_EM_ (C), and T_E_ (D) of individual replicates at the various time points are shown as a percent of all OVA_257–264_-specific CD8^+^ T cells. At different time points after infection an *in vivo* CTL assay was performed in representative mice (n = 3 mice per time point per group), (E). Frequency of IFN-γ producing OVA_257–264_-specific CD8^+^ T cells in the spleen (F), liver (G) and inguinal lymph nodes (H) at different time points after infection is shown. Data in panels F–G represent Mean ± SD of triplicate ELISPOT wells of n = 2 mice per group. This experiment was repeated 3 independent times with similar results.

Naïve CD8^+^ T cells express high levels of IL-7Rα and CD62L. Once activated CD8^+^ T cells down regulate both receptors, later on in the memory phase, IL-7Rα is again up-regulated and CD62L expression may be variable depending on the type of immunization [22], [23]. Effector memory cells (T_EM_) are IL-7Rα^high^CD62L^low^ whereas central memory cells (T_CM_) are IL-7Rα^high^CD62L^high^, and the latter phenotype cells afford long-lasting tumor protection [3]. Therefore we assessed if qualitative differences existed between the antigen-specific CD8^+^ T cells evoked in WT and FtDKO mice. On day 7 after LM-OVA infection OVA_257–264_-specific CD8^+^ T cells had a similar low level expression of IL-7Rα and CD62L in both mouse strains (Fig. 3A) indicative of an effector response. In the memory phase as well, 93 days after infection, the up-regulation of IL-7Rα and CD62L on OVA_257–264_-specific CD8^+^ T cells occurred similarly in both strains of mice (Fig. 3A). At day 101, 8 days after tumor challenge, the OVA_257–264_-specific CD8^+^ T cell population down-regulated IL-7Rα and CD62L indicating a return to a predominant effector phenotype. Furthermore, the proportion of T_CM_ (Fig. 3B), T_EM_ (Fig. 3C) and CD62L^low^IL-7Rα^low^ effector (Fig. 3D) CD8^+^ T cells was comparable between WT and FtDKO at each time point. Thus, FtDKO mice induced CD8^+^ T cells of similar kinetics and phenotypic proportions as that of WT during both the effector and memory phases of the immune response.

### LM-OVA induces functional OVA-CD8^+^ T cells in FtDKO mice

Selectin ligands such as PSGL-1 may be induced on CD8^+^ T cells upon activation [8]. Therefore, we next sought to investigate whether there was any difference in the functionality of selectin ligand-deficient FtDKO OVA_257–264_-specific CD8^+^ T cells upon activation. Within 7 days of infection both WT and FtDKO mice eliminated nearly 100% of target cells in vivo. Over time, the killing of target cells waned equally in both strains of mice indicating contraction of the CD8^+^ T cell response (Fig. 3E). Similarly, the frequency of IFN-γ secreting CD8^+^ T cells was similar in both strains of mice in the different tissues (Figs. 3F, G & H). However, due to low numbers of lymph node cells in FtDKO mice at later time points, CD8^+^ T cell frequencies were not ascertained in this tissue beyond 50 days. Overall, the lack of long-term tumor protection in FtDKO mice could not be attributed to non-functional CD8^+^ T cell response.

### FtDKO mice effectively control primary LM-OVA infection, induce a strong recall CD8^+^ T cell response and are protected against a bacterial re-challenge

**Figure 4:**
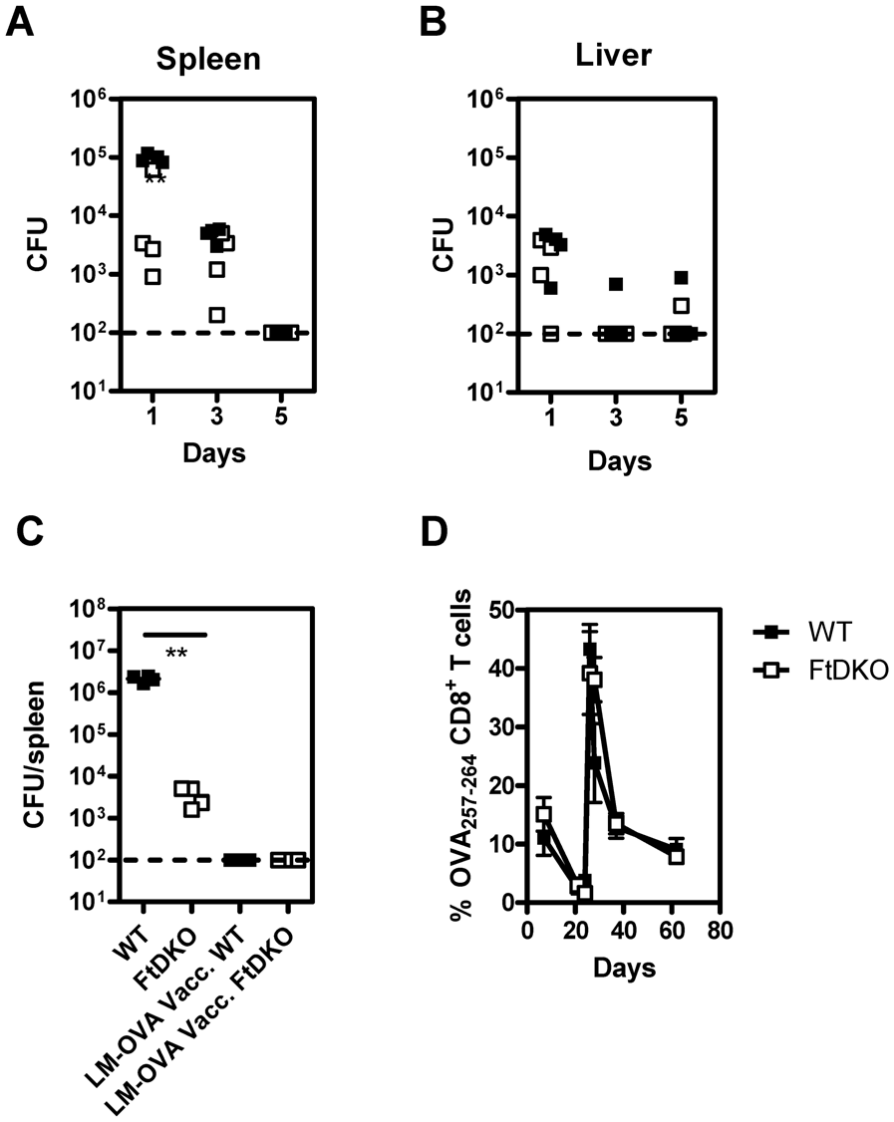
LM-OVA clearance and re-challenge response. Bacterial burden in the spleen (A), and liver (B) of individual mice on different days post-infection (n = 4/time point/group) following intravenous 10^4^ LM-OVA infection. **, P = 0.0029 by two-tailed *t* test with Welch's correction for unequal variance. In a separate experiment, mice vaccinated with 10^4^ LM-OVA were re-challenged 21 days later with 10^5^ LM-OVA, (C & D). Splenic bacterial burden on day 3 following 10^5^ LM-OVA infection (C), n = 4/group. **, P = 0.0013 relative to C57BL/6J by two tailed *t*-test with Welch's correction for unequal variance. The frequency of blood OVA_257–264_-specific CD8^+^ T cells was determined (D) following primary exposure to 10^4^ LM-OVA and re-challenge on day 21 with 10^5^ LM-OVA (n = 3/group).

We next ascertained if LM-OVA infection itself was differentially controlled in FtDKO mice. Bacterial burden was enumerated in the spleen and liver following intravenous infection with 10^4^ LM-OVA (Fig. 4A and B). A diminished splenic bacterial burden was noted in FtDKO mice relative to wild-type on day 1 post-infection whereas no differences in liver bacterial burden were observed at any time point. This may be attributable to the increased numbers of neutrophils and monocytes in the spleens of FtDKO mice relative to other organs that occurs due to lymph node atrophy. Overall, both strains of mice cleared LM-OVA infection classically within 5 days. In a separate experiment WT mice were challenged with a lower dose of LM-OVA (10^3^ instead of 10^4^ CFU) resulting in a lower bacterial burden similar to FtDKO mice 1 day after infection. 21 days after infection they were challenged with B16-OVA; again WT mice were afforded longer lasting protection from tumor compared to FtDKO mice vaccinated with a higher dose (Fig. S2).

In order to test the recall ability of OVA_257–264_-specific CD8^+^ T cells following initial priming, a re-challenge infection of 10^5^ LM-OVA was done 21 days after initial vaccination. As observed above, non-vaccinated FtDKO mice had a significantly decreased splenic bacterial burden compared to WT (Fig. 4C) even to a higher dose (10^5^) of LM-OVA. However, in vaccinated mice, both FtDKO and WT had undetectable splenic LM-OVA burden upon re-challenge indicating a rapid secondary response. Concurrently, the LM-OVA re-challenge caused the rapid expansion of blood resident OVA_257–264_-specific CD8^+^ T cells in both mouse strains indicative of a strong memory recall response (Fig. 4D). Thus, while FtDKO mice exhibited diminished protection against tumor challenge, their CD8^+^ T cell response was effective against infection re-challenge.

### Dendritic cells (DCs) of FtDKO mice induce strong antigen presentation to CD8^+^ T cells

**Figure 5:**
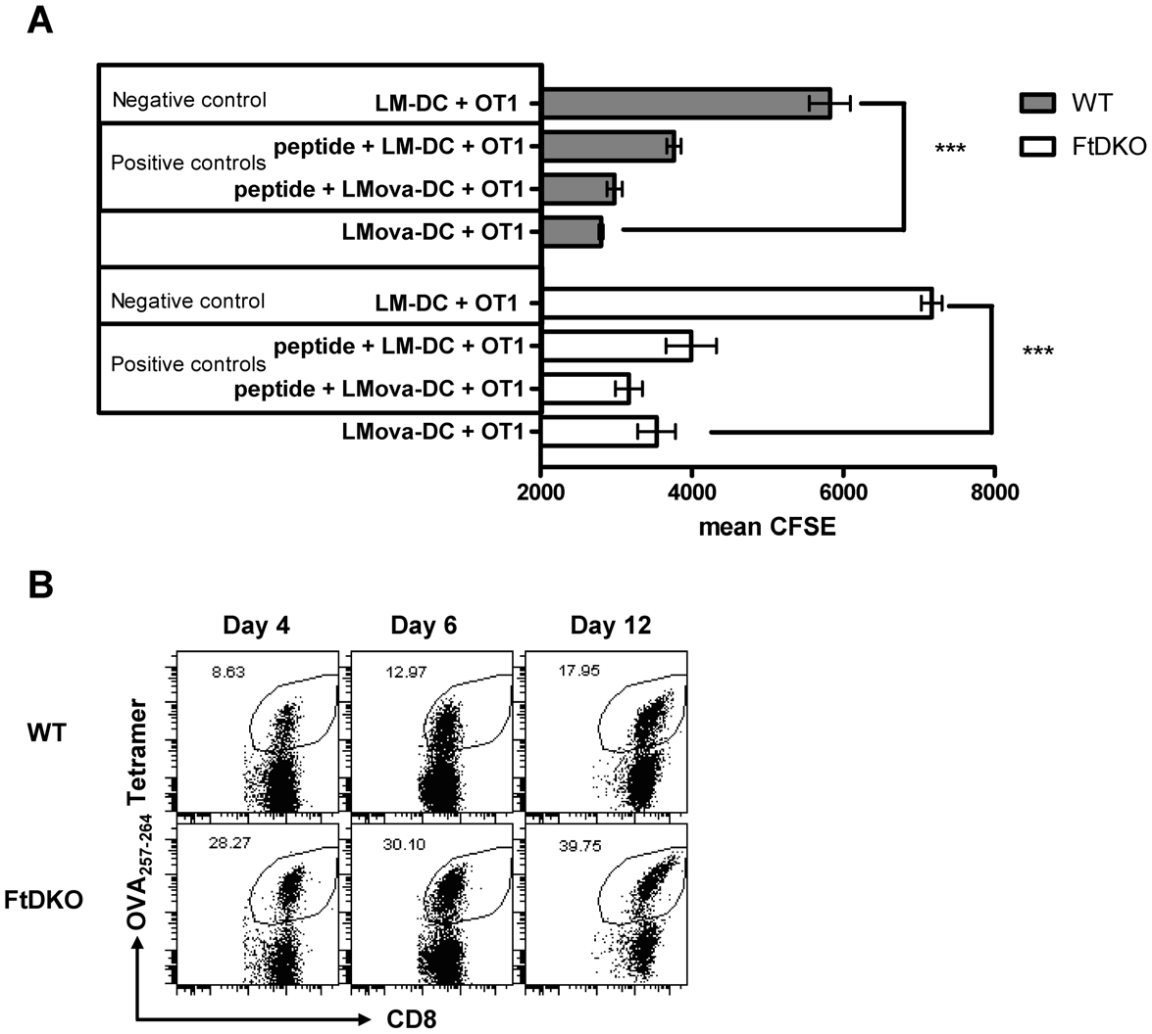
Antigen presentation and proliferation. Mean ± SD of triplicate cultures of CFSE reduction correlates to proliferation of OT.1 CD8^+^ T cells in response to DCs from LM or LM-OVA infected mice. Positive controls were pulsed with OVA_257–264_ peptide in vitro. Data are significantly different, ***(p<0.0001), relative to negative control (LM infected DCs) by two tailed *t* test with Welch's correction for unequal variance. Data are representative of 2 independent DC isolation experiments. (B) Representative flow cytometric profile indicating in-vitro antigen-dependent proliferation of OVA_257–264_-specific CD8^+^ T cells on different days of in vitro culture. Samples were obtained from in-vitro cultures in T-75 flasks. Data are representative of three such independent experiments conducted.

Next, to investigate any potential defect in antigen presentation, we measured the ability of splenic DCs from LM-OVA infected mice to present OVA_257–264_ antigen to CD8^+^ T cells ex-vivo. Successful antigen presentation resulted in robust CD8^+^ T cell proliferation and CFSE dilution (Fig. 5A). DC's from LM infected mice lacking in-vivo exposure to antigen served as negative controls and OVA_257–264_ peptide pulsed DC's served as positive controls. DC's from FtDKO and WT mice infected with LM-OVA were capable of inducing CD8^+^ T cell proliferation similar to positive controls (Fig. 5A) as well as rapid CD62L down-regulation (data not shown). Therefore no intrinsic antigen-presentation defect in the DCs of FtDKO mice was discerned.

The superior ability of T_CM_ cells to contain tumor growth is attributed to their proliferative ability. Hence we determined if CD8^+^ T memory cells from FtDKO mice may have reduced ability to rapidly proliferate in response to antigen. Splenic cultures from vaccinated mice stimulated in vitro with LM-OVA and cytokines, and analyzed 4, 6 and 12 days later exhibited a steady expansion of antigen-specific CD8^+^ T cells over time (Fig. 5B). Moreover, greater number of antigen-specific CD8^+^ T cells was noted for FtDKO cells relative to WT cultures. Subsequently, the number of OVA_257–264_-specific CD8^+^ T cells increased in both groups. Thus, the lack of selectin ligands on CD8^+^ T cells did not impair their proliferation.

### The infiltration of OVA-specific CD8^+^ T cells into the draining lymph nodes and tumors of FtDKO mice is impaired

**Figure 6:**
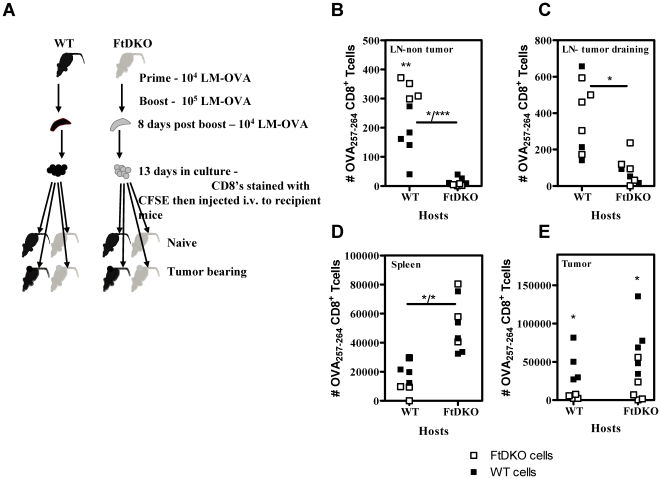
Migration of Adoptively transferred antigen-specific CD8^+^ T cells. Adoptive transfer experiment was carried as indicated in the schematic diagram (A). Purified CD8^+^ T cells from in-vitro antigen-dependant expansion of cultures (comprising 1×10^6^ OVA_257–264_-specific CD8^+^ T cells) were transferred intravenously into naïve or tumor bearing recipient mice (tumor size, 45–90 mm^2^) , and donor cells in the various organs of recipients were enumerated 4 days later. Number of OVA_257–264_-specific CD8^+^ T cells in individual mice recovered from non-tumor bearing lymph nodes (B), tumor bearing lymph nodes (C) tumor bearing spleen (D) and tumor (E) of recipient WT or FtDKO mice is indicated. (B), *, WT cells in FtDKO compared to WT hosts, p = 0.02; ***, FtDKO cells in FtDKO compared to WT hosts, p = 0.0003; **, FtDKO cells compared to WT cells in WT hosts; p = 0.008. (C), *, FtDKO cells in different hosts, p = 0.011. (D), *, WT cells in different hosts, p = 0.03; * FtDKO cells in different hosts, p = 0.036. (E), *, WT cells compared to FtDKO cells in WT hosts, p = 0.034; *, FtDKO versus WT cells in FtDKO hosts, p = 0.043. Data were analyzed by unpaired *t* test.

Since FtDKO mice displayed normal antigen-presentation, CD8^+^ T cell expansion, contraction and function, we next analyzed the tumor infiltrative properties of adoptively transferred CD8^+^ T cells. CD8^+^ T cells were expanded in vitro from vaccinated mice, and then adoptively transferred into non-tumor and tumor bearing recipients (Fig. 6A). Transfer of equal number of OVA-specific CD8^+^ T cells was done in mice 9 days after tumor challenge, and mice harbored palpable tumors in the range of 45–90 mm^2^. Firstly, significantly greater numbers of OVA_257–264_-specific CD8^+^ T cells infiltrated the lymph nodes of WT compared to FtDKO recipients (Fig. 6B and C). This trend was noted for both non-tumor (Fig. 6B) and tumor bearing (Fig. 6C) recipients and for WT or FtDKO donor cells. In contrast FtDKO recipients harbored more donor cells (WT or FtDKO) in the spleen (Fig. 6D). In WT recipients, FtDKO and WT donor cells migrated to the lymph node and spleen at similar levels (Fig. 6B, C & D). Therefore, the FtDKO host environment appeared to skew the trafficking of donor cells away from the lymph node compartment. Furthermore, significantly lower numbers of FtDKO donor cells were recovered from the tumor tissue of both WT and FtDKO recipients (Fig. 6E). However, high numbers of donor WT cells were found in tumors of both WT and FtDKO hosts. Taken together, these results indicate a defect in homing of antigen specific CD8^+^ T cells (WT or FtDKO) to the lymph nodes of FtDKO recipient mice and compromised migration of functional selectin ligand-deficient CD8^+^ T cells into the subcutaneous tumor.

**Figure 7:**
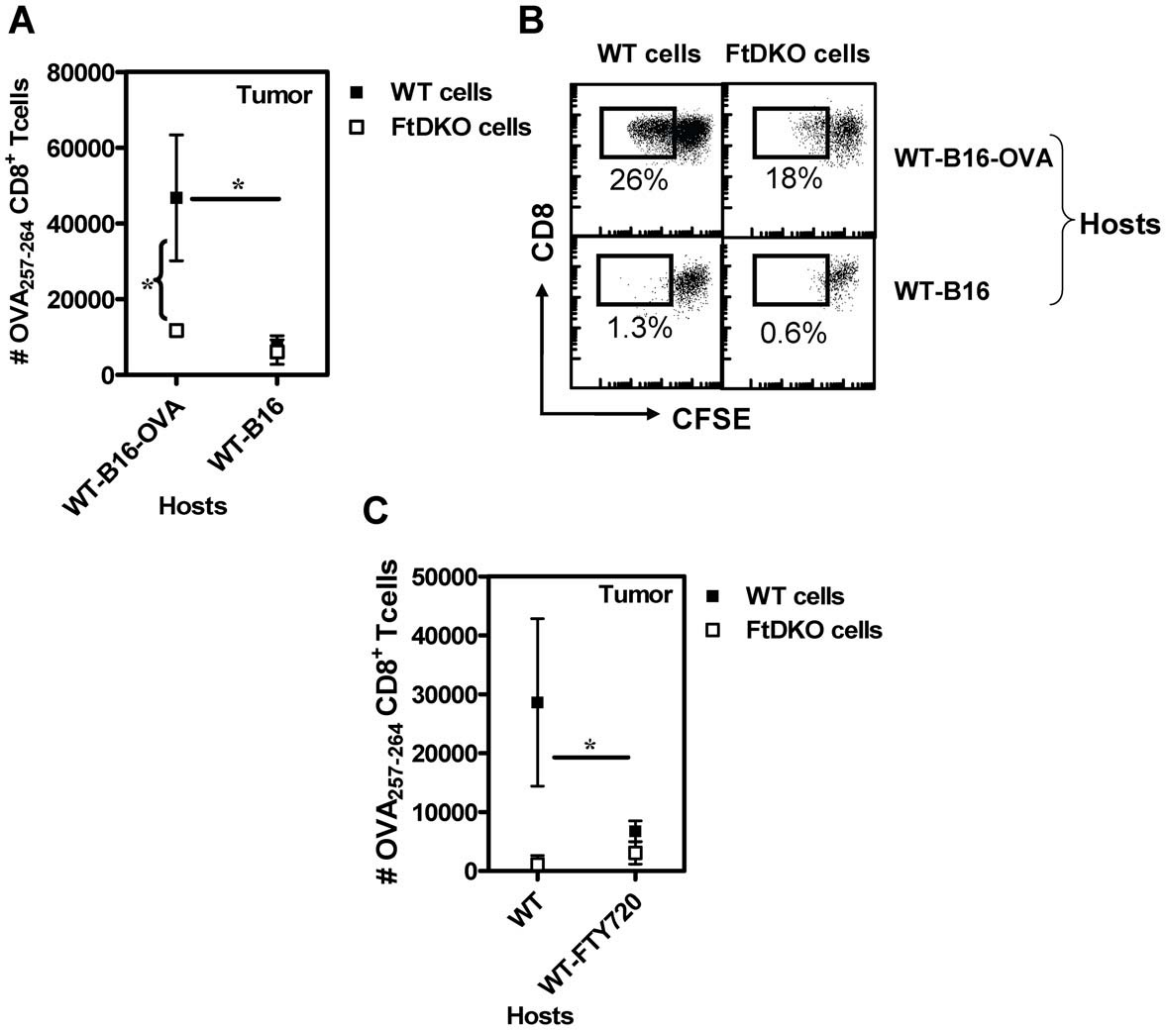
Effect of tumor antigen and lymph node egress in the migration of adoptively transferred antigen-specific CD8^+^ T cells. Adoptive transfer experiment was carried as indicated in the schematic diagram in Fig. 6A. CFSE-labeled, 1.5×10^6^ OVA_257–264_-specific CD8^+^ T cells (obtained from in-vitro expanded cultures) were transferred intravenously into day 9 tumor bearing mice (mean tumor size 45–90 mm^2^). Mice were euthanized 4 days later and the number of donor cells in the various organs of recipients was enumerated. (A), Mean ± SD (n = 3–5/group) of OVA_257–264_-specific CD8^+^ T cells (of WT or FtDKO origin) recovered from the tumor tissue of WT hosts bearing B16-OVA or B16 tumors respectively is indicated. *, WT cells in B16-OVA compared to B16 tumor tissue, p<0.05. *, WT cells versus FtDKO cells in B16-OVA tumor tissue, p<0.05. (B), Representative flow cytometric profile of tumor-infiltrating CD8^+^ T cells demonstrating CFSE dilution in WT hosts bearing B16-OVA tumors. (C), Mean ± SD (n = 3–4/group) of OVA_257–264_-specific CD8^+^ T cells (of WT or FtDKO origin) recovered from the tumor tissue of WT hosts that are untreated on treated with FTY720. *, WT cells in WT hosts compared to WT cells in WT-FTY720 treated hosts, p<0.05.

Next, we addressed if the lower numbers of FtDKO donor cells homing to WT tumor was antigen dependant. When OVA-specific WT or FtDKO donor CD8^+^ T cells were transferred into WT hosts bearing B16 tumors, there was no migration seen to the tumor indicating that movement of adoptively transferred cells into the tumor was antigen-dependant (Fig. 7A). Moreover, as expected the donor cells that migrated to B16-OVA tumor exhibited antigen-dependant proliferation as evidenced by dilution of CFSE (Fig. 7B). Finally, in order to elucidate the migration path of donor CD8^+^ T cells into the tumor tissue, we carried out adoptive transfer experiments in conjunction with in vivo FTY720 treatment of recipients. FTY720 is a known chemical inhibitor that blocks egress of lymphoid cells from the lymph node [24]. Fig. 7C shows that in FTY720-treated recipients, there was a dramatic decrease in the number of donor WT CD8^+^ T cells migrating into the tumor, indicating that tumor infiltrating donor cells egress from the draining lymph node of the recipient. Taken together with observations in Fig. 6E, these data suggest that in the absence of functional selectin ligands on CD8^+^ T cells, the egress of cells from lymph node into the peripheral tumor is impaired.

## Discussion

Adhesion events that occur between antigen-specific CD8^+^ T cells and their environment may influence their initial activation, maintenance as memory cells, reactivation upon antigenic encounter, and migration to the tumor site. Among the many adhesion molecules involved in cell adherence, selectin-selectin ligand interactions occur early on and mediate rolling and tethering of lymphocytes along the endothelium. We therefore surmised that modulation of such mechanisms in FtDKO mice lacking selectin ligands [25]–[27] may influence tumor surveillance by CD8^+^ T cells. Induced expression of adhesion molecules such as chemokine receptors, integrins or selectins on T cells has been reported to enhance their migration to the periphery [28]–[30]. Furthermore, reducing the expression of the late adhesion molecule, VLA-4 on CD8^+^ T cells, reduces their recruitment into tumors [31]. In many cancer models the infiltration of CD8^+^ T cells into a tumor site correlates with improved prognosis [32]–[34]. Therefore, understanding the events that draw tumor reactive CD8^+^ T cells into a tumor environment is vital for the development of efficacious anti-tumor vaccines.

We have previously shown that LM-OVA vaccination evokes T_CM_ cells and consequent long-term protection against B16-OVA melanoma [13], providing a convenient model to evaluate the tenets of anti-tumor immunity. Interestingly, FtDKO mice exhibit similar kinetics of clearance of *Mycobacterium tuberculosis* and Lymphocytic choriomeningitis virus infections in visceral organs [19], [35], despite a disparate frequency of CD8^+^ T cells in the lymph node but similar frequencies in other organs [20], [35], [36]. In contrast, FtDKO mice had significantly decreased splenic burden of LM-OVA as early as 1 day after infection. However, this did not reduce efficient antigen-presentation by splenic DCs of FtDKO mice (Fig. 5A). Furthermore, FtDKO mice had severely enlarged spleens (Fig. S1) possibly due to the increased numbers of monocytes and neutrophils [17], [19]. Moreover, 7 days after LM-OVA infection, FtDKO mice had significantly higher frequencies of antigen-specific CD8^+^ T cells in the blood. Overall, the enhanced innate immune response combined with an uncompromised CD8^+^ T cell response in the spleens and livers of FtDKO mice may have contributed to accelerated clearance of LM-OVA that shows natural tropism for spleen and liver.

The presence of the selectin, CD62L, on antigen experienced CD8^+^ T cells (T_CM_) correlates with superior tumor protection [10], [12], [37]. CD62L is capable of binding a number of cell associated selectin ligands including glycosylated cell adhesion molecule-1, CD34, mucosal addressin cell adhesion molecule-1, sulphated glycoprotein 200, and P-selectin glycoprotein ligand-1 [36], [38]. Mice singly deficient in α(1,3) Fucosyltransferase-VII (FucTVII^−/−^) have reduced cell numbers in lymph nodes [17] as well as reduced CD4^+^ and CD8^+^ T cell homing into the skin as shown by a contact hypersensitivity assay [27], [36]. However FucTVII^−/−^ mice exhibit an incomplete inhibition of E-selectin ligand formation. Complete inhibition of selectin ligand binding including E-selectin interactions occurs in FtDKO mice doubly deficient for α(1,3) Fucosyltransferase-IV and VII. In this model, selectin ligand interactions have been credited to mediate effector CD8^+^ T cell recruitment to the myocardium [26] as well as naive and memory CD8^+^ T cell entry into lymph nodes [19]. We have shown that despite the normal ability of FtDKO mice to mount a classic acute immune response to LM-OVA, including induction of CD62L^high^ T_CM_ cells, their ability to mediate immune surveillance against a growing tumor is compromised. Thus, the interaction of CD62L on CD8^+^ T cells with its fucosylated ligands appears to be important for their ability to infiltrate peripheral tumors.

In a previous study, Vesicular stomatitis virus (VSV) vaccinated singly deficient FucTVII^−/−^ mice challenged intravenously 14 days later with EL-4N1 (a T cell tumor cell line expressing the viral nucleoprotein N1) exhibited similar protection as wild-type mice [36]. However in that study, protection was reported only up to 50 days post tumor challenge. Similarly, we observed that following intravenous challenge with B16-OVA, FtDKO mice were protected similar to WT controls when observed a few weeks after tumor challenge. However, later on FtDKO mice succumbed to tumors at a faster rate relative to controls. Nevertheless, the protection against metastatic tumor challenge was less severely compromised in FtDKO mice. This may be attributed to previous observations that T cell homing to non-lymphoid organs such as lung and liver remains efficient in the absence of selectin ligand interactions.

Vaccinated FtDKO mice succumbed to tumors at a faster pace than WT mice when challenged early (day 21) or late (day 126) post-vaccination. This suggests that the compromised tumor immunity may not be simply attributed to ineffective maintenance of the CD8^+^ T cell response. Additionally, vaccinated FtDKO mice exhibited a delay in tumor development relative to non-vaccinated controls. Thus, selectin ligand deficiency does not appear to fully negate short-term effector function of antigen-specific CD8^+^ T cells. However, the expression of selectin ligands is induced on some activated CD8^+^ T cells and can play a role in their extravasation into sites of injury by binding to P-selectin and E-selectin [18]. Our observation that following adoptive cell transfer, antigen-specific FtDKO CD8^+^ T cells were recovered in substantially lower numbers in the tumors of WT recipients suggests that selectin ligand deficient CD8^+^ T cells may have an intrinsic extravasation defect. This agrees with an earlier study showing fewer FucTVII^−/−^ OVA-specific OT-1 CD8^+^ T cells entering an inflamed footpad compared to WT [26]. However, the low numbers of FtDKO cells at the tumor site may correlate to a reduced retention and/or replenishment of these cells over time. Indeed, lymphoid proliferation of T cells and then peripheral migration may be required for sustained tumor killing. Schuster et al [39] showed that naïve antigen-specific CD62L^−/−^ T cells adoptively transferred to a RAG^−/−^ host bearing a MC57-SIY-IND fibrosarcoma were defective in responding to tumor antigen and affording protection relative to CD62L^+/+^ T cells. Selectin ligand interactions can influence naive T cell trafficking to lymph nodes [27], [40]. However recently it was postulated that antigen-experienced CD8^+^ T cells can traffic to lymphoid compartments via other non-high endothelial venules such as afferent lymph [19]. Harp et al reported reduced T cell egress from FtDKO lymph nodes results in enrichment of CD44^high^ CD8^+^ T memory cells [19] possibly attributable to differential recirculation of lymphocytes in a reduced cell density environment [41]. Overall, circulating CD8^+^ T cells may have directly migrated to highly vascularized B16-OVA tumors via the blood vasculature, utilizing selectin ligand independent interactions, thus providing some short-term protection. However, our results demonstrate that preventing egress of even WT antigen-specific CD8^+^ T cells from lymph nodes substantially reduces their numbers in tumors. The homing of T cells to the skin has been shown to be partially dependent on CCR4 [42]. However, we observed that the expression of CCR4 was similar in WT and FtDKO CD8^+^ T cells at all times after LM-OVA vaccination (data not shown). Thus, the absence of selectin ligands alone on the FtDKO CD8^+^ T cells may have had a secondary consequence of abrogating sustained egress of these cells from the lymph node into the peripheral tumor, compromising long-term protection. Overall, the presence of selectin ligands on both the host high endothelial venules and CD8^+^ T cells may be important for efficient and continued extravasation and/or retention within peripheral tumors such as melanoma.

In the last decade, adoptive T cell transfer-based cancer immunotherapy has gained prominence in clinical trials including the use of genetically engineered T lymphocytes that express high affinity T cell receptors of known cancer antigen specificity [43]–[45]. Furthermore, focus is shifting towards refining the qualities in a candidate anti-tumor T cell, such as increased homeostatic proliferation and increased expression of co-stimulatory molecule CD27 along with reduced expression of regulatory molecules such as KLRG-1, CD57 and Eomes such that tumor evasive properties are effectively counteracted while optimizing the functionality of anti-tumor T cells [37]. The optimization of selectin ligand interactions on CD8^+^ T cells in such adoptive transfer T cell preparations may be yet another important criterion for the maintenance of long-term anti-tumor immunity.

## Materials and Methods

### Ethics Statement

All animals were housed in the animal facility of the National Research Council-Institute for Biological Science and maintained according to the guidelines of the Canadian Council of Animal Care (CCAC). Protocols and procedures (Protocol # 2008.24) were approved and monitored by the National Research Council of Canada-Institute for Biological Sciences Animal Care Committee.

### Bacterial strain and assessment of bacterial burden

LM-OVA was grown as previously described [46]. Spleen and liver of infected mice were homogenized in 0.9% NaCl and colony forming units (CFU's) were determined by plating 100 µl of serial 10-fold dilutions on BHI agar plates (Difco laboratories).

### Mice and immunizations

C57BL/6J (WT) mice were obtained from The Jackson Laboratory. FucT-IV/-VII-deficient (FtDKO) mice on a C57BL/6J genetic background were generated by Dr. John Lowe [25] and obtained through the Consortium for Functional Glycomics, and bred subsequently in-house by homozygote matings. For immunization frozen LM-OVA bacterial stocks were thawed and diluted in 0.9% NaCl. Female mice were inoculated with 1×10^4^ organisms (unless otherwise indicated) suspended in 200 µl of 0.9% NaCl, via the lateral tail vein.

### Murine Melanoma model

B16 and B16-OVA (expressing plasmid derived ovalbumin) cells were obtained from Dr. Edith Lord (University of Rochester, Rochester, NY). B16-OVA cells were cultured in RPMI 1640 medium supplemented with 8% Fetal Bovine Serum (FBS) containing 400 µg/ml of G418 (for plasmid antibiotic selection) and 10 µg/ml of gentamicin. B16-OVA cells (1×10^6^ in PBS+0.5% normal mouse serum) were injected subcutaneously in the shaven lower dorsal region of mice. From day 5 onwards, detectable solid tumor was measured using calipers. Tumor size, expressed in mm^2^, was obtained by multiplication of diametrically perpendicular measurements. End-points for the survival graphs were based on a maximum tumor size of 200 mm^2^. For the metastatic model, B16-OVA cells (5×10^5^) were injected intravenously via the lateral tail vein. Mice were euthanized 15 days later for enumeration of black lung tumor foci. Conversely, survival was determined based on 20% loss in body weight or visible clinical signs of illness such as piloerection, hunched posture and lethargy.

### Assessment of phenotype of OVA_257–264_-specific CD8^+^ T cells

At various time intervals after infection, 50 µl aliquots of whole blood was incubated with anti-CD8 (clone 53-6.7, BD Pharmingen), anti-CD62L (clone MEL-14, BD Pharmingen), anti-IL-7Rα (CD127, clone A7R34, BD Pharmingen) antibody and H-2K^b^OVA_257–264_ tetramer (Beckman Coulter) diluted in PBS plus 1% bovine serum albumin (PBS-BSA) for 30 min. Red blood cells were lysed and cells were washed with PBS, fixed in 1% formaldehyde, and acquired on BD Biosciences FACS Canto analyzer.

### Assessment of CD8^+^ T cell function

*In vivo* cytolytic activity of antigen-specific CD8^+^ T cells was enumerated as described previously [47]. Briefly, infected recipient mice were injected with a 1∶1 mixture of differentially labeled OVA_257–264_ peptide-target and control cells. The survival proportions of donor cell populations in recipient spleens were determined 24 h later, and percentage *in vivo* killing calculated. Enumeration of IFN-γ-secreting cells was performed by ELISPOT assay [46]. Liver lymphocytes were obtained after tissue homogenization and percoll (GE Healthcare Biosciences) density gradient centrifugation. Lymphocytes (at a final density of 5×10^5^ cells/well by using feeder splenocytes) were incubated on anti-IFN-γ-antibody-coated ELISPOT plates and stimulated with OVA_257–264_ peptide (10 µg/ml) in RPMI+8% FBS containing IL-2 (0.01 ng/ml), for 48 h at 37°C, 8% CO_2_. Plates were then washed (0.01% Tween in PBS) and incubated with the biotinylated secondary antibody (2 h at 37°C), followed by streptavidin-horse radish peroxidase conjugate (1 h at room temperature). Spots were developed using AEC (3-amino-9-ethylcarbazole) peroxidase substrate solution.

### CD8^+^ T cell primary cultures and adoptive transfer experiments

Splenocytes (10^8^) pooled from infected mice (n = 2–3) were stimulated with 10^4^ LM-OVA in-vitro and cultured in 10 ml RPMI+8% FBS in T-25 tissue culture flasks (Falcon), at 37°C and 8% CO_2_. Gentamicin (50 µg/ml) was added to the cultures 15 h later to kill any live LM-OVA. Subsequently at 40 h after in-vitro antigen exposure, cells were transferred in fresh media supplemented with IL-7 and IL-15 (1 ng/ml) to a T-75 tissue culture flask and thereafter expanded with media and cytokine supplementation every two days. For analysis of proliferation, a sample of cells was retrieved on various days after culture and analyzed for the expression of OVA_257–264_-tetramer-specific CD8^+^ T cells by flow cytometry. For adoptive transfer experiments, CD8^+^ T cells were purified from day 13 cultures expanded as detailed above using CD8^+^ T cell negative enrichment kit (STEMCELL Inc.). Equal number of antigen-specific CD8^+^ T cells (deduced from flow cytometric analysis of in-vitro cultures) of either WT or FtDKO phenotype were injected (i.v.) into naïve or day 9 tumor bearing animals. Day 9 tumor bearing mice had tumor sizes in the range of 45–90 mm^2^.

### FTY720 treatment

Mice were injected intraperitoneally (i.p.) with 1 mg/Kg FTY720 (Selleck Chemicals) in 200 µl (diluted in PBS after dissolution of the compound in a small volume of DMSO) 24 h after adoptive transfer of antigen-specific CD8^+^ T cells and then every 24 h until euthanasia of mice and harvest on day 4 after adoptive transfer.

### Dendritic cell purification and assessment of antigen presentation

Spleen cells were obtained from mice 1 day after 10^4^ LM or LM-OVA infection and treated with spleen dissociation medium (STEMCELL Inc.). Dendritic cells (DC's) were purified using the positive selection DC purification kit (STEMCELL Inc.) that consistently, 80–84% purity. DCs (3×10^5^/well) were co-cultured for 4 days with CFSE (0.125 µM) stained splenocytes obtained from OT.1 mice (10^5^ per well), in u-bottom 96-well microtiter plates, in RPMI+8% FBS and 50 µg/ml gentamicin. OT.1 mice are transgenic for the TCR of OVA and harbor >99% OVA-specific CD8^+^ T cells in their lymphoid organ. Positive controls included cells cultured with 5 µg/mL of OVA_257–264_ peptide. Proliferation of CD8^+^ T cells was measured 4 days later by assessing CFSE reduction by flow cytometry.

## Supporting Information

Figure S1 **Mice (WT and FtDKO) were infected (i.v.) with 10^4^ LM-OVA.** Spleens were isolated at varying time points after infection and enumerated for numbers of lymphocytes by microscopy with trypan blue staining. n = 3–4 mice/group/time-point.
(TIF)

Figure S2 **Mice (WT and FtDKO) were infected (i.v.) with 10^3^ or 10^4^ LM-OVA respectively.** (A), Spleens were removed on day 1, 3 or 5 after infection and bacterial burden was determined (n = 3–4 mice). (B), another group of infected mice were challenged with 10^6^ B16-OVA and survival based on a maximum tumor size of 300 mm^2^ was monitored. Data is compiled from two separate studies. Median survival of vaccinated WT mice was 68 days, and 39 days for FtDKO mice. n = 4–5 mice per group.
(TIF)
