# Supplementary figures and images for: Lack of Functional Selectin Ligand Interactions Compromises Long Term Tumor Protection by CD8+ T Cells
Source: PLoS One. 2012 Feb 16;7(2):e32211. doi: 10.1371/journal.pone.0032211 (PMC3281134; doi:10.1371/journal.pone.0032211)

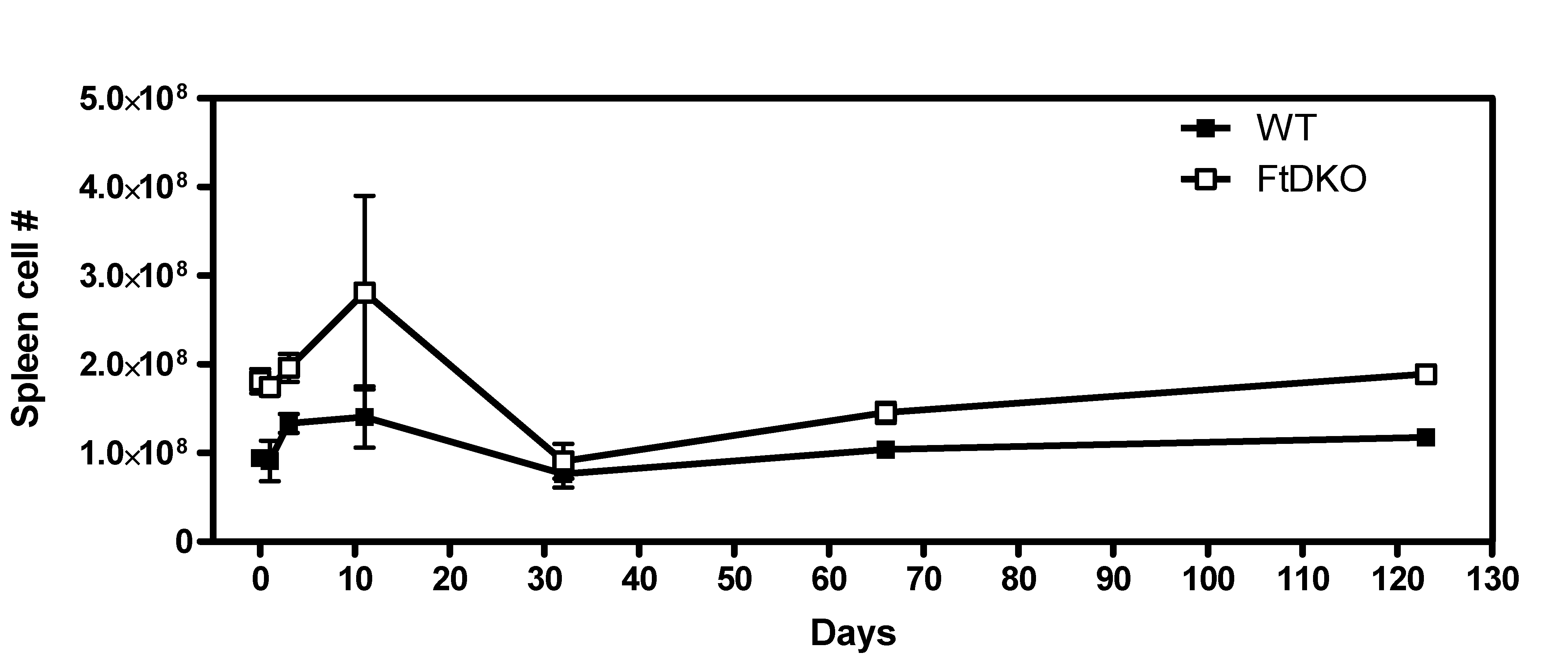

Supplement: Figure S1 — Mice (WT and FtDKO) were infected (i.v.) with 104 LM-OVA. Spleens were isolated at varying time points after infection and enumerated for numbers of lymphocytes by microscopy with trypan blue staining. n = 3–4 mice/group/time-point. (TIF) [file pone.0032211.s001.tif]

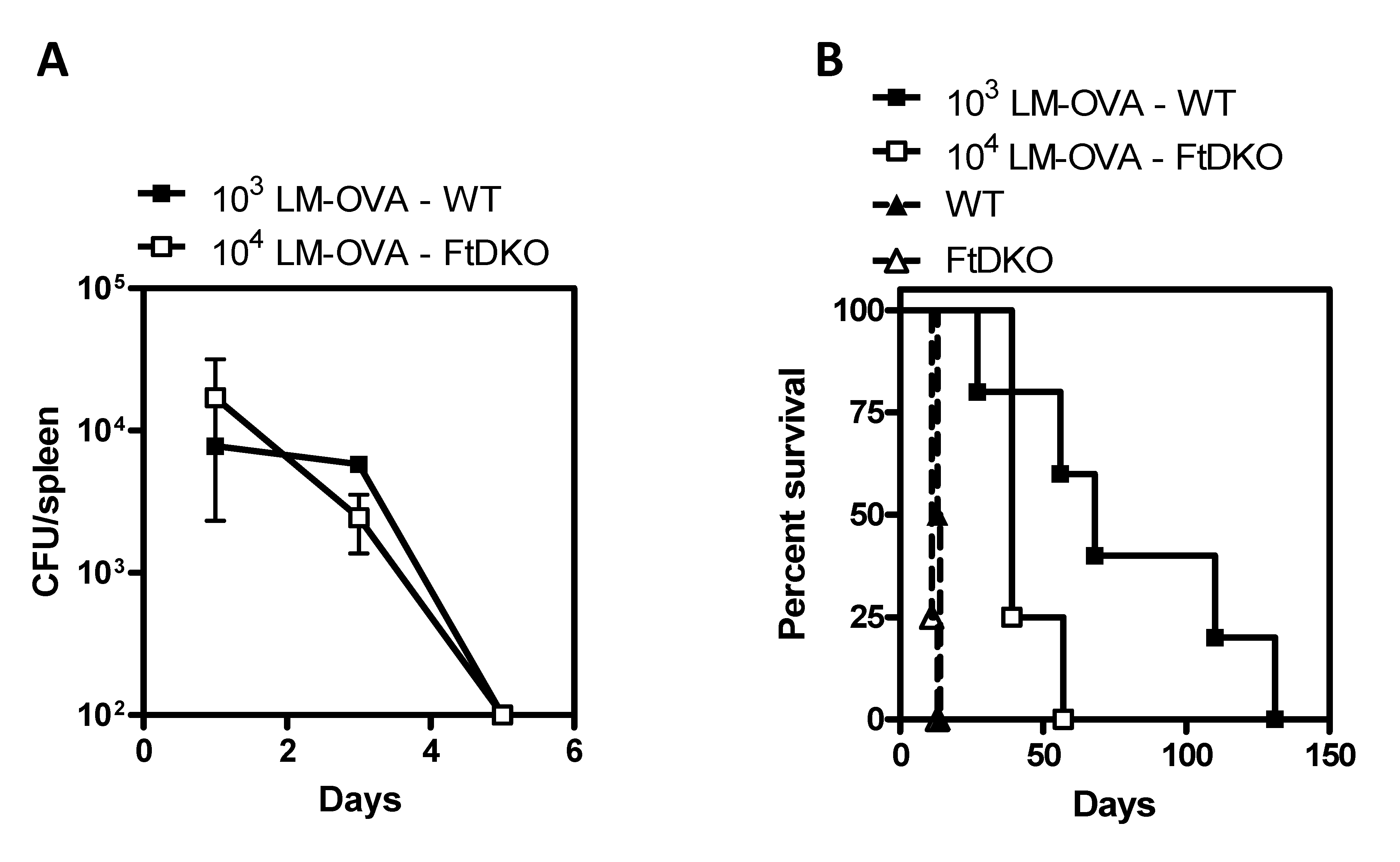

Supplement: Figure S2 — Mice (WT and FtDKO) were infected (i.v.) with 103 or 104 LM-OVA respectively. (A), Spleens were removed on day 1, 3 or 5 after infection and bacterial burden was determined (n = 3–4 mice). (B), another group of infected mice were challenged with 106 B16-OVA and survival based on a maximum tumor size of 300 mm2 was monitored. Data is compiled from two separate studies. Median survival of vaccinated WT mice was 68 days, and 39 days for FtDKO mice. n = 4–5 mice per group. (TIF) [file pone.0032211.s002.tif]
